# The biogeography of gastrointestinal mucosal microbiota of beef cattle at harvest

**DOI:** 10.3389/fmicb.2024.1490882

**Published:** 2024-12-09

**Authors:** J. Daniel Young, Lee J. Pinnell, Cory A. Wolfe, Enrique Doster, Robert Valeris-Chacin, Ty E. Lawrence, John T. Richeson, Paul S. Morley

**Affiliations:** ^1^Department of Agricultural Sciences, West Texas A&M University, Canyon, TX, United States; ^2^VERO Program, Texas A&M University, Canyon, TX, United States

**Keywords:** 16S, microbiome, feedlot, sequencing, qPCR

## Abstract

**Introduction:**

The gastrointestinal microbiota profoundly influences the health and productivity of animals. This study aimed to characterize microbial community structures of the mouth, gastrointestinal tract (GIT), and feces of cattle.

**Methods:**

Samples were collected from 18 Akaushi crossbred steers at harvest from multiple locations, including the oral cavity, rumen, abomasum, duodenum, jejunum, ileum, cecum, spiral colon, distal colon, and feces. These cattle were raised without exposure to antimicrobial drugs or hormone implants. Total microbial abundance was assessed using qPCR targeting the V3–V4 region of the 16S rRNA gene, and microbial community composition was evaluated through 16S rRNA gene sequencing.

**Results:**

Total microbial abundance was lesser in the small intestine than in other GIT regions (*p* ≤ 0.05). Additionally, microbial communities in the small intestine had lower richness and diversity than other regions (*p* ≤ 0.05). Microbial community compositions were measurably different along the GIT, with greater relatedness in adjacent GIT sections when progressing from oral to aboral locations. Firmicutes, Bacteroidota, and Actinobacteria were the dominant phyla in all samples. However, variations in composition were evident at lower taxonomic levels within these dominant phyla among samples from different regions. Genera previously associated with healthy gut microbiome communities were observed in low abundance across GIT regions. Taxa historically associated with liver abscesses (e.g., *Fusobacterium* and *Trueperella*) were detected in low abundance (≤0.02% relative abundance) throughout the GIT. In contrast, *Bacteroides*, which recently has been identified as a dominant feature in many liver abscesses, was observed in greater relative abundance (5.2% on average) in the hindgut.

**Discussion:**

This study provides an in-depth evaluation of the GIT of harvest-ready Akaushi crossbred cattle of varying growth rates. Clear differences exist in the abundance and composition of microbial populations at different points of the GIT. Unfortunately, no single GIT location can adequately represent the microbial communities of the entire GIT, which has important implications for future research. Additionally, examining microbiome data only at the phylum level likely oversimplifies important complexities of the microbial community structures, and investigations of lower taxonomic ranks should be included.

## 1 Introduction

The gastrointestinal tract (GIT) is a complex organ system essential for multiple vital functions in animals, as in people. Investigations of the biogeography of the GIT in people have identified fascinating relationships with metabolic function and health (McCallum and Tropini, 2023). However, research characterizing microbial community composition across all GIT regions in feedlot cattle remains limited, and there is insufficient evidence supporting the validity of extrapolating findings from other cattle types.

While microbial populations in the rumen and feces have been extensively studied (de Oliveira et al., 2013; Holman and Gzyl, 2019; Guo et al., 2020; Lin et al., 2023) investigations are lacking regarding other parts of the GIT, especially in high-marbling breeds that are finish-fed in North American feedlots. The few studies that have explored the biogeography of gut microbiota have studied a limited number of GIT locations and have used limited, albeit variable, sequencing depth (de Oliveira et al., 2013; Mao et al., 2015; Plaizier et al., 2020). Notably, a meta-analysis by Holman and Gzyl (2019) summarizing 52 prior investigations concluded that greater knowledge is needed regarding microbiomes of the small intestine and colon in cattle.

There is a growing impetus to use feed supplements in cattle in an attempt to modify the microbiome and convey health or performance benefits (Welch et al., 2022). But there is a need to characterize the GIT microbiome constituents and functions to provide logical, context for evaluating the utility of these products (Kinross et al., 2011). Further, creating a baseline of normal GIT microflora will provide valuable comparisons for future work exploring shifts in microbial dynamics in diseased animals.

The primary objective of our research was to investigate the composition of gut microbial communities at multiple locations throughout the GIT in harvest-ready high-marbling feedlot steers.

## 2 Materials and methods

### 2.1 Study overview

The mouth, GIT, and feces of 18 feedlot cattle harvested in two cohorts representing animals with greater and lower feed efficiencies were sampled to characterize the biogeography of gut microbiota. Swab samples were collected from 10 locations including the mouth, rumen, abomasum, duodenum, jejunum, ileum, cecum, spiral colon, distal colon, and feces. Since liver abscesses have been linked to decreased performance, purulent material from liver abscesses was also sampled if present. After extraction and purification from swabs, the total microbial abundance of samples was assessed using qPCR targeting the 16S rRNA gene (16S qPCR), and the diversity and composition of microbial communities were characterized using 16S rRNA gene sequencing of the V3–V4 region (16S sequencing). All antemortem procedures used in cattle rearing were approved by the West Texas A&M University Institutional Animal Care and Use Committee – Protocol# 15.99.05.W1.02AR.

### 2.2 Study population and sample collection

Eighteen Akaushi cross-bred steers enrolled in a production program that certifies the absence of exposure to antimicrobial drugs and exogenous hormones were managed at the West Texas A&M University Research Feedlot. Cattle received corn-based rations and had no antimicrobial drug or exogenous hormone exposures at any time during their lives. Cattle were housed in 6 × 26 m dirt-surfaced pens and provided *ad libitum* access to water, but no shade was provided. Cattle were fed once daily at 0730. The average body weight at enrollment was 381 kg (95% Confidence Interval (95%CI): 356, 407 kg). Cattle were fed high-concentrate rations in small confinement pens (4 pens of 5 animals; 2 animals died before harvest and were not sampled). The larger cattle at feedlot arrival (Cohort 1) were split into 2 pens, as were the smaller cattle (Cohort 2). After 56 d on feed, these cattle were transitioned to a finishing ration (Supplementary Table S1). Cattle remained on this ration until harvest. All cattle were enrolled in August 2021 but were split into two harvest groups with Cohort 1 harvested in May 2022, and Cohort 2 harvested in July 2022. The more rapidly growing animals (*n* = 9) were harvested at 271 d on feed, and the second group (*n* = 9) was harvested 78 days later at 349 d on feed. Average daily gains were 0.74 kg/d (95%CI: 0.62, 0.86 kg/d) for the first cohort and 0.63 kg/d (95%CI: 0.51, 0.75 kg/d) for the second. When cattle reached an acceptable level of body condition for harvest (i.e., visually appraised to have approximately 1.27 cm (0.5 in) of subcutaneous rib fat), they were harvested at the West Texas A&M University Caviness Meat Science & Innovation Center (USDA Est. #7124). The average live weight at harvest was 591 kg (95%CI: 560, 623 kg) with an average hot carcass weight of 366 kg (95%CI: 348, 385 kg) and an average dressing percent of 63.3% (95%CI: 61.4, 65.2%). All cattle were classified as A maturity with an average ribeye area of 84.28 cm^2^ (95%CI: 79.73, 88.79 cm^2^) and an average marbling score of Small^89^ (95%CI: Small^48^, Modest^29^). Liver abscesses were identified in 3 animals, one of which had two abscesses (2 animals in Cohort 1 and 1 animal in Cohort 2).

Before transport from the feedlot to the harvest facility, cattle were individually weighed, oral rayon-tipped swabs were used to sample the buccal region (20.3 cm long, 1.3 cm tip diameter; Puritan, Guilford, ME), and feces were collected per rectum using gloves that were changed between animals. At the harvest facility, cattle were rendered senseless using a captive bolt gun, and USDA-approved, industry-standard procedures were used for harvest of beef products. Evisceration occurred within 20 min of initial stunning, and the entire gastrointestinal tract was placed on a stainless-steel table in a room separated from the harvest floor. Gut segments were identified, and a ~2.5 cm long, full-thickness incision was created at standardized locations using a new sterile disposable scalpel (Cynamed, Lorton, VA) for every incision. These incisions were held open with sterile forceps, and a sterile rayon-tipped swab (Puritan) was inserted to collect samples from the mucosal surfaces. Swab samples were then placed into sterile conical tubes and immediately placed upon ice. After samples had been collected from the rumen, abomasum, duodenum, jejunum, ileum, cecum, spiral colon, and distal colon of each animal, gut tissues were removed from the room, and the table was cleaned with water, dried, and then sanitized with a cleaning solution (RNAse Away; Molecular BioProducts, San Diego, CA) between animals.

Within 4 h of collection, samples were transported to the research laboratory at the Texas A&M University VERO building. All samples were stored at −80°C until further processing.

### 2.3 DNA isolation

DNA was isolated from all samples using a commercial extraction kit (QIAamp Power Fecal Pro DNA, Qiagen, Hilden, Germany) and an automated nucleic acid extraction system (Qiacube Connect, Qiagen), following the manufacturer’s recommendations. DNA was quantified (ng/μL) using fluorometry (Qubit Flex, Thermo Fisher Scientific). Every batch (11 samples per batch) of extractions processed included an extraction blank, which was included in the downstream library preparation and sequencing.

### 2.4 qPCR to quantify the total microbial abundance

We used qPCR targeting the V3–V4 region of the 16S rRNA gene as a proxy assessment for total microbial abundance in samples. A total of 173 samples had sufficient DNA to be analyzed with 16S rRNA gene sequencing and 16S qPCR. Extraction yielded limited amounts of DNA from some samples, and 16S sequencing was prioritized for those samples. One sample that failed amplification was also removed from the qPCR investigation. Samples were evaluated by qPCR in triplicate, and the 20 μL final reaction contained 10 ng of sample DNA, 1X Quantabio PerfeCTa SYBR Green FastMix, and 450 nM of each primer [341F/785R, forward: CCTACGGGNGGCWGCAG, reverse: GACTACHVGGGTATCTAATCC; (Klindworth et al., 2013)]. Serial dilutions of purified *Mannheimia haemolytica* genomic DNA were used to create a standard curve ranging from 20 million to 20 copies of the bacterial 16S rRNA gene. *Mannheimia haemolytica* serial dilutions were run simultaneously with samples to quantify bacterial abundance. Thermal cycling was performed with a QuantStudio™ 3 Real-Time PCR system (QS3; Applied Biosystems, Thermo Fisher Scientific). Cycling conditions were as follows: 2 min at 50°C for UDG activation, followed by denaturation at 95°C for 10 min, and then 40 cycles of 95°C for 15 s, and 50°C at 15 s 58°C. The process finished with the melt curve stage at 95°C for 15 s, 58°C for 30 s, and a 0.15°C/s ramp to 95°C, with a hold at that temperature for 1 s.

### 2.5 16S rRNA gene sequencing

The V3–V4 region of the 16S rRNA gene was amplified using the 341F/785R primer pair as previously described (Klindworth et al., 2013). DNA amplification steps were conducted at 98°C for 3 min, 98°C for 30 s for 18 cycles, 72°C for 1 min, and then 72°C for 5 min. Amplicon libraries were prepared according to Illumina’s protocol (Illumina Inc., 2013) and pooled for sequencing equimolarly. The resulting pooled library was sequenced using an Illumina NovaSeq 6,000 instrument using 2 × 250 base pair (bp) paired-end chemistry at the Texas A&M Institute for Genome Sciences and Society sequencing core. Negative controls, extraction blanks, and no-template PCR controls (nuclease-free water) were included but did not yield any detectable product and consequently were removed from downstream statistical analysis.

### 2.6 Bioinformatics

Demultiplexed sequencing reads were imported into QIIME2 version 2023.2 (Bolyen et al., 2019). Amplicon sequencing variants (ASVs) were generated using DADA2 (Callahan et al., 2016), which also filters for read quality, removes chimeric sequences, and merges overlapping paired-end reads. Forward reads were trimmed at 17 bp and reverse reads at 21 bp, and all reads were truncated at 249 bp. Taxonomy was assigned using a Naïve Bayes classifier trained on the SILVA 138.1 SSU NR 99 database (Quast et al., 2012) where sequences had been trimmed to only include base pairs from the V3–V4 region delimited by the 341F/785R primer pair. Reads mapping to chloroplast and mitochondria were removed from the ASV table and representative sequences. A mid-point rooted phylogenic tree was created using ‘qiime alignment mafft,’ ‘qiime alignment mask,’ and ‘qiime phylogeny fasttree’ under default settings. The ASV table, representative sequences, and mid-point rooted tree were imported into phyloseq (McMurdie and Holmes, 2013) using the ‘import_biom’ function. Using the ‘import_qiime_sample_data’ function, metadata was imported and merged with the ASV table, representative sequences, and tree into a phyloseq object.

Richness (observed numbers of ASVs) and Faith’s observed phylogenic distance were calculated using the ‘estimate_richness’ and ‘estimate pd’ functions of the phyloseq and btools packages, respectively. Then, ASV counts were normalized using cumulative sum scaling (Paulson et al., 2013). Beta diversity was analyzed using generalized UniFrac distances (Chen et al., 2012; Paulson et al., 2013). Using these distances, non-metric multidimensional scaling (NMDS) was employed for plotting, and permutational multivariate analysis of variance (PERMANOVA) was used to test for differences in community structure using the ‘vegan’ (Oksanen et al., 2022) and ‘pairwiseAdonis’ (Martinez Arizu, 2020) packages. Additionally, a permutational analysis of dispersion (PERMDISP) was conducted for all significant PERMANOVA’s to ensure the differences were not generated from unequal dispersions of variance between groups. Using the ‘hclust’ function, hierarchical clustering was performed using Ward’s agglomeration method (Murtagh and Legendre, 2014) on the generalized UniFrac distances and plotted with the ggdendro package to generate dendrograms. Further, relative abundances were calculated and plotted using phyloseq. Specific taxa of interest were identified *a priori* and subset using phyloseq. A pairwise Wilcoxon rank-sum analysis of variance was used to determine the difference in their relative abundance between locations along the GIT.

### 2.7 Biologically important taxa and core microbiome analysis

The relative abundance of specific taxa known or believed to be important in feedlot cattle and the Firmicutes to Bacteroidota (F:B) ratios were calculated for samples collected at the different GIT locations. The F:B ratio was calculated by dividing Firmicutes’ relative abundance (RA) by the RA of Bacteroidota in each sample. Biologically important taxa identified *a priori* were further investigated individually using the ‘subset taxa’ function in the phyloseq package in R. Bacterial genera of interest were *Bacillus, Bifidobacterium, Lactobacillus, Fusobacterium, Trueperella, Bacteroides,* and *Porphyromonas*. These bacteria were specifically explored because of previous evidence that they are linked to improved health or increased disease risks (Nagaraja and Chengappa, 1998; Binda et al., 2018; Smock et al., 2020; Cull et al., 2022; Pinnell and Morley, 2022). Additionally, the entire class of Gamma-proteobacteria was explored because of the significant pathogenic potential of some members, including *Salmonella* and *Escherichia-Shigella*. Core members of the microbiome were characterized at the genus level using the microbiome package in R, and a minimum detection threshold was set at 0.1% RA, with the minimum prevalence set at 90%.

### 2.8 Statistical analysis

Statistical analysis was conducted using R version 4.2.2 (Team, 2020). All qPCR data were tested for normality using the Shapiro–Wilk test. Because the data were not normally distributed, a Wilcoxon pairwise rank-sum test with a Benjamini–Hochberg false discovery rate (FDR) correction was used to test for differences. For univariate microbiome data (i.e., alpha diversity metrics, individual taxa RA values), a Kruskal–Wallis analysis of variance was used for the comparison between 2 variables or a Wilcoxon pairwise rank-sum test with a Benjamini-Hochberg FDR correction for multiple comparisons between more than two variables. For multivariate comparisons (i.e., beta diversity), differences were tested using pairwise PERMANOVA with a Benjamini–Hochberg FDR correction for multiple comparisons and 9,999 permutations. Additionally, when appropriate, pairwise PERMDISPs were used with 9,999 permutations to test for differences in the variability of dispersions. Differences were considered statistically significantly different, when appropriate, using a critical *α* cutpoint of 0.05.

## 3 Results

### 3.1 Total microbial abundance

**Figure 1:**
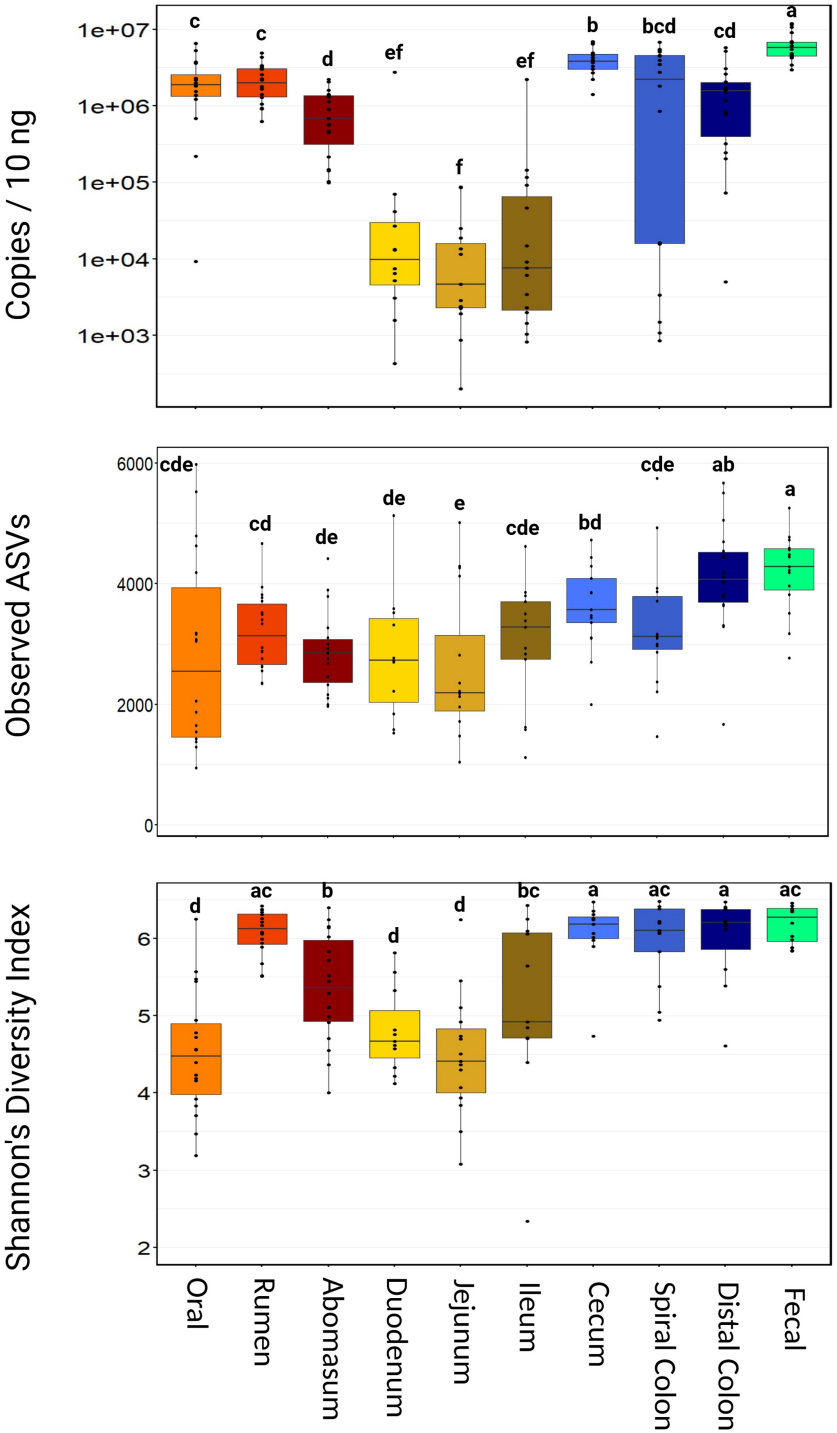
Boxplot of total microbial abundance and Alpha diversity by sampling location. Boxes with different superscripts (abcdefg) differ by pairwise Wilcoxon ranked sum test, (*p* ≤ 0.05). Plots were created in R, and legends were added using BioRender.com.

Using 16S qPCR, the average microbial abundance among all samples was 2,110,275 copies per 10 ng of input DNA. There was no difference in the microbial abundance between harvest groups (*p* = 0.34; Supplementary Figure S1). However, microbial abundance varied widely throughout the GIT (Figure 1). Fecal samples had the highest microbial abundance (mean = 6,422,873 copies/10 ng, range = 2,953,871–11,979,323 copies/10 ng, *p* < 0.001). Oral, rumen, spiral colon, and distal colon samples were intermediate in microbial abundance relative to other sampling locations. Small intestine samples (duodenum, jejunum, and ileum) all had lower microbial abundance compared to the other GIT samples (*p* ≤ 0.05).

### 3.2 Sequencing metrics

Samples with fewer than 300,000 ASVs per sample (*n* = 13) were removed from downstream analysis. The remaining samples (*n* = 163) averaged 1,267,216 ASVs per sample (range = 312,234–2,363,766 ASVs). Rarefaction curves demonstrated that this sequencing depth was adequate to detect all members of the bacterial communities that can be characterized using 16S rRNA amplicon sequencing (Supplementary Figure S2). Notably, ASV richness increased in a significant proportion of samples when subsampled to <250,000 PE reads and did not plateau for all samples until approximately 750,000 to 1,000,000 PE reads. There was no difference in the number of ASVs identified between animals from either harvest group (*p* > 0.24). Nearly all ASVs (>99%) could be classified at the ranks of phylum, class, order, and family, and approximately 93% of ASVs were classified at the genus level (Supplementary Table S2).

### 3.3 Alpha diversity

Richness and diversity were different between harvest cohorts (*p* < 0.001), with group 2 demonstrating more observed ASVs and significantly greater Shannon’s diversity index (Supplementary Figure S3). Richness also varied considerably across sampling locations, with the abomasum, duodenum, and jejunum all having fewer (*p* ≤ 0.05) observed ASVs than the cecum, distal colon, and fecal samples (Figure 1). Shannon’s diversity index was also significantly different among sample locations. The oral, duodenum, and jejunum samples were less diverse (*p* ≤ 0.05) than the rumen, cecum, spiral colon, and fecal samples.

### 3.4 Beta diversity

**Figure 2:**
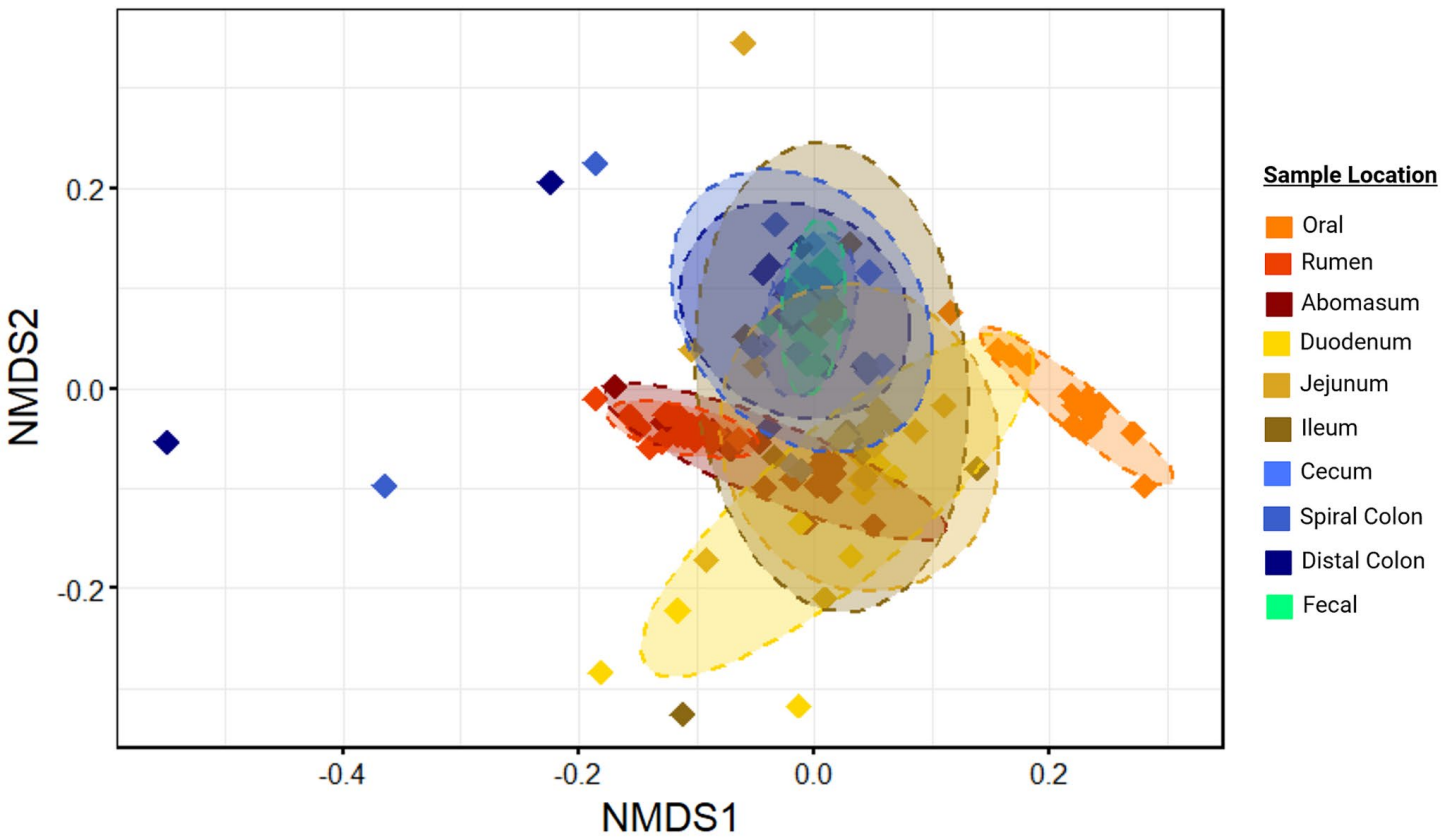
Ordination of community structure by sample location. Non-metric multidirectional scaling (NMDS) of generalized Unifrac distances illustrate differences in microbial community structure between sample sites. Ellipses represent the 95% confidence intervals for the group mean values. Plots were created in R, and legends were added using BioRender.com.

Microbial communities from different harvest cohorts, and animals demonstrated significantly different compositions (*p* < 0.01; *r*^2^ = 0.02 and 0.17 respectively, Supplementary Figure S4), as did communities from different GIT locations based on generalized Unifrac distances (*p* ≤ 0.05; *r*^2^ = 0.38; Figure 2). However, a statistically significant PERMDISP statistic for comparison of GIT location suggested unequal dispersion of variances, which may have inflated the Type I error in this comparison.

**Figure 3:**
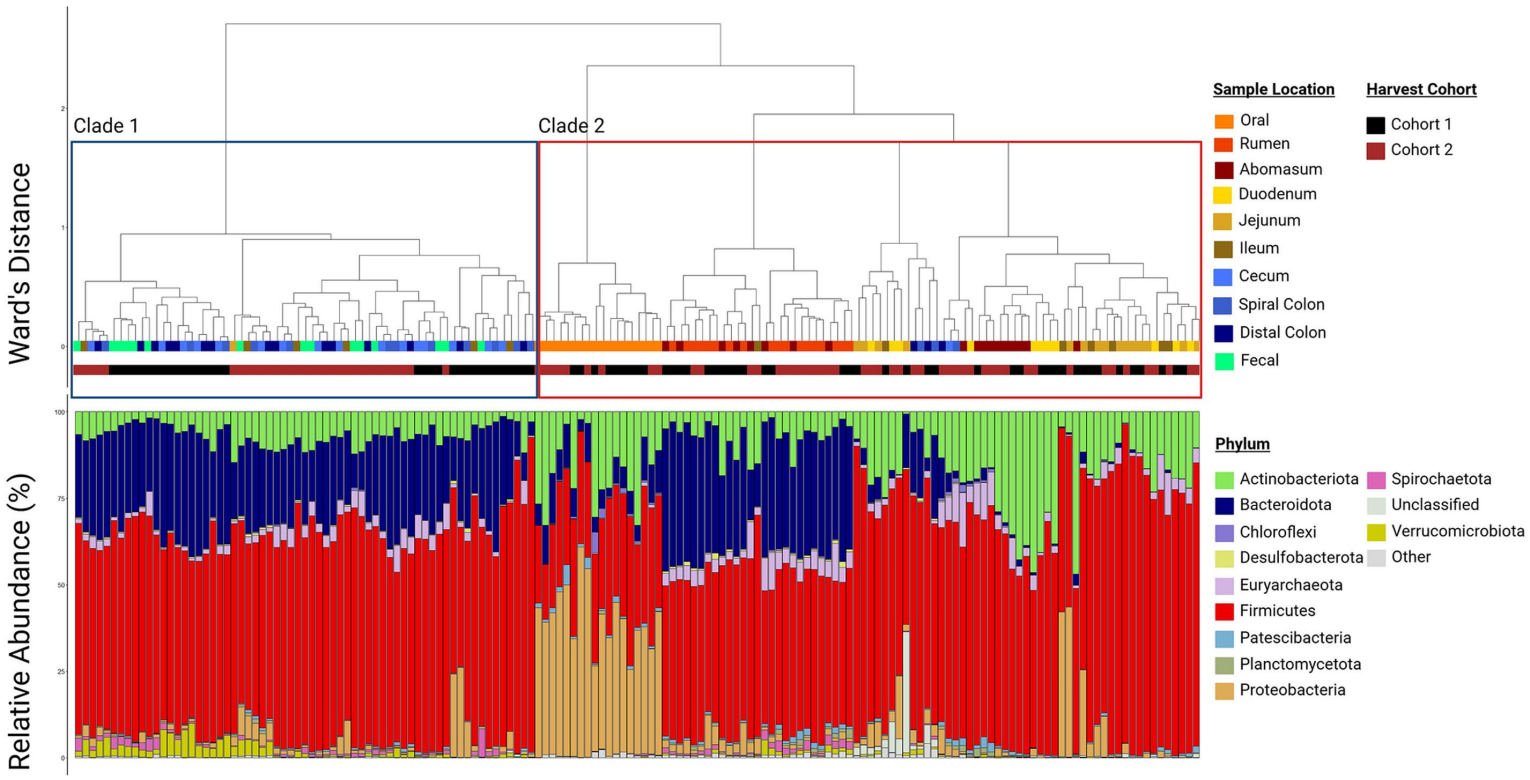
Paired dendrogram and relative abundance plot by sample location. Clustering of similar sample sites reinforces that samples from similar locations have similar community structures and the relative abundance plot shows which phyla compose those communities. Each box in the relative abundance plot is an individual sample and corresponds to the dendrogram above denoting what type of sample is being represented. Plots were created in R, and legends were added using BioRender.com.

Regardless, there was clear evidence that the oral samples clustered separately in the ordination plots (Figure 2). This supports the conclusion that this community structure differed from other locations in the GIT. Other sample locations showed more similarity to anatomically adjacent sites than to more separated locations (e.g., rumen and abomasum, abomasum and small intestine sites, ileum and colon, large intestine sites and feces), as evidenced by overlapping 95% confidence ellipses. Thus, there was an apparent transition in microbial community composition and structure from proximal to distal GIT ends. This was reinforced by the hierarchical clustering of microbial communities (Figure 3). Two distinct clades were identified in the dendrogram, with most hindgut samples clustering in clade 1 and the remaining samples clustering in clade 2.

**Figure 4:**
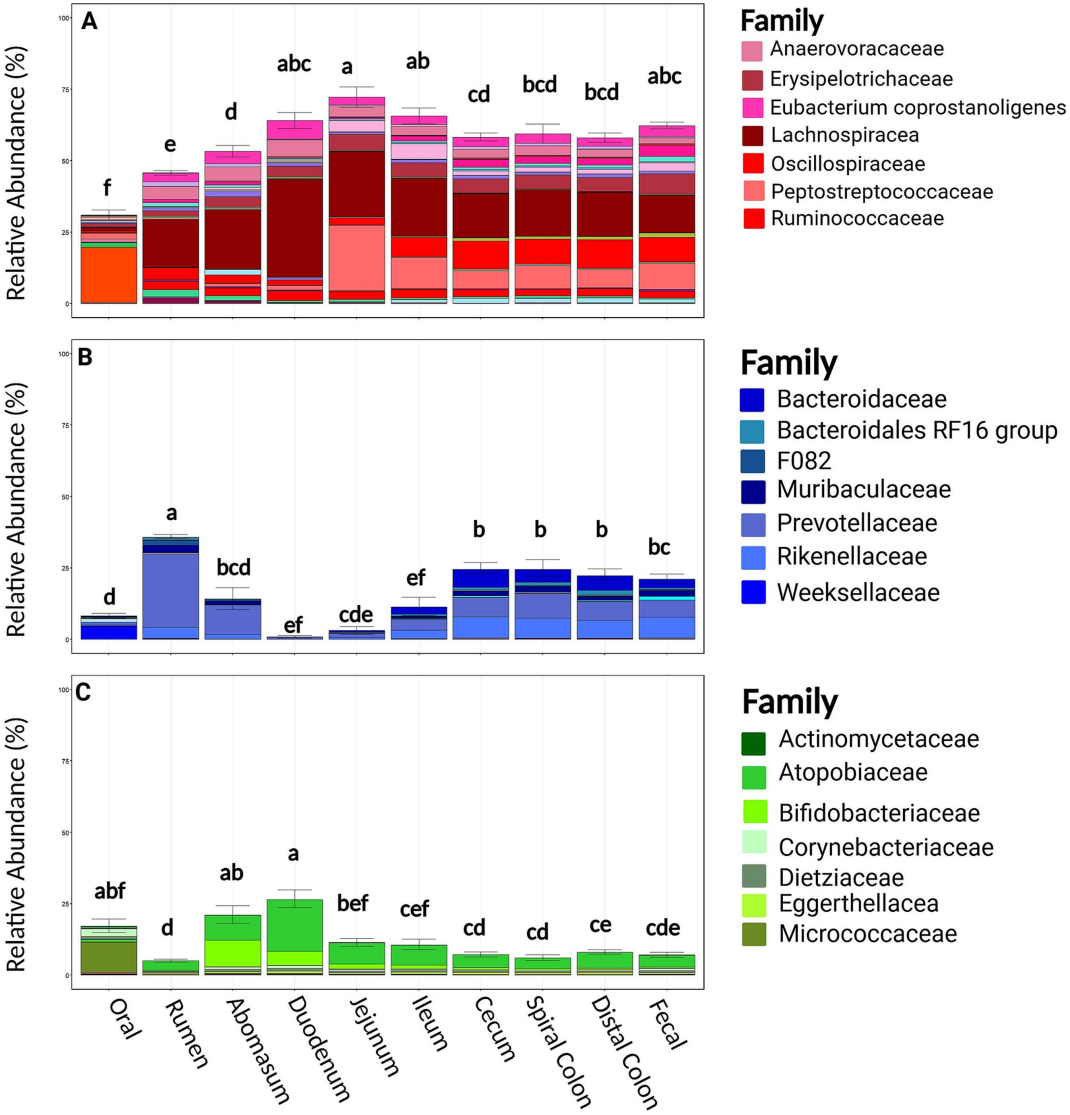
Relevant phyla by family and sample location. Panel **(A)** represents families belonging to the Firmicutes phyla split by sample site. Panel **(B)** represents families belonging to the Bacteroidota phyla split by sample site. Panel **(C)** represents families belonging to the Actinobacteriota phyla split by sample site. Within a figure, sample sites with differing superscripts (abcdefg) differ by pairwise Wilcoxon rank sum test (*p* ≤ 0.05). Plots were created in R, and legends were added using BioRender.com.

At the phylum level, Firmicutes, Bacteroidota, and Actinobacteriota were the most common phyla at all sampling locations in both harvest cohorts [mean RA 54.8%, (95%CI: 52.2, 57.3%); mean 17.6%, (95%CI: 15.5, 19.61%); mean 11.9% (95%CI: 10.3, 13.4%); respectively]. Families within the phylum Firmicutes were relatively diverse; a total of 118 bacterial families were identified, and the 7 most abundant comprised 41.3% of taxonomical classifications (*Lachnospiraceae, Peptostreptococcaceae, Oscillospiraceae, Erysipelotrichaceae, Anaerovoracaceae, [Eubacterium] coprostanoligenes group*, and *Ruminococcaceae*; Figure 4). Bacteroidota was less rich, being comprised of 57 families, but the seven most abundant families comprised only 16.3% of the observed abundance (*Prevotellaceae, Rikenellaceae, Bacteroidaceae, Muribaculaceae, Bacteroidales RF16, Weeksellaceae,* and *F082*). Actinobacteriota was similarly less rich with 73 families, with the seven most abundant families representing 11.1% of the relative abundance within this phylum (*Atopobiaceae, Bifidobacteriaceae, Micrococcaceae, Corynebacteriaceae, Dietziaceae, Eggerthellaceae,* and *Actinomycetaceae*). The RA of these three dominant phyla were variable between harvest cohorts and sampling locations and correspondingly were associated with differences in the community structures and clustering of data of these samples (Supplementary Figure S4; Figure 2). Firmicutes and Bacteriodota RA were not different between harvest cohorts (*p* ≥ 0.22). Firmicutes RA was the greatest in the duodenum, jejunum, and ileum but lowest in the oral, rumen, and abomasum samples (*p* ≤ 0.05, Figure 4). In contrast, Bacteroidota RA was the greatest in the rumen and significantly greater in the cecum spiral colon and distal colon when compared to the duodenum, jejunum, and ileum (*p* ≤ 0.05). Actinobacteria was different across harvest cohorts (*p* = 0.001, Supplementary Figure S4). Notably, cohort 1 contained greater *Bifidobacteria* and less *Atopobiaceae* than cohort 2. Regionally, Actinobacteria RA was the greatest in the abomasum and duodenum and the lowest in the rumen (*p* < 0.001).

### 3.5 Biologically important taxa

**Figure 5:**
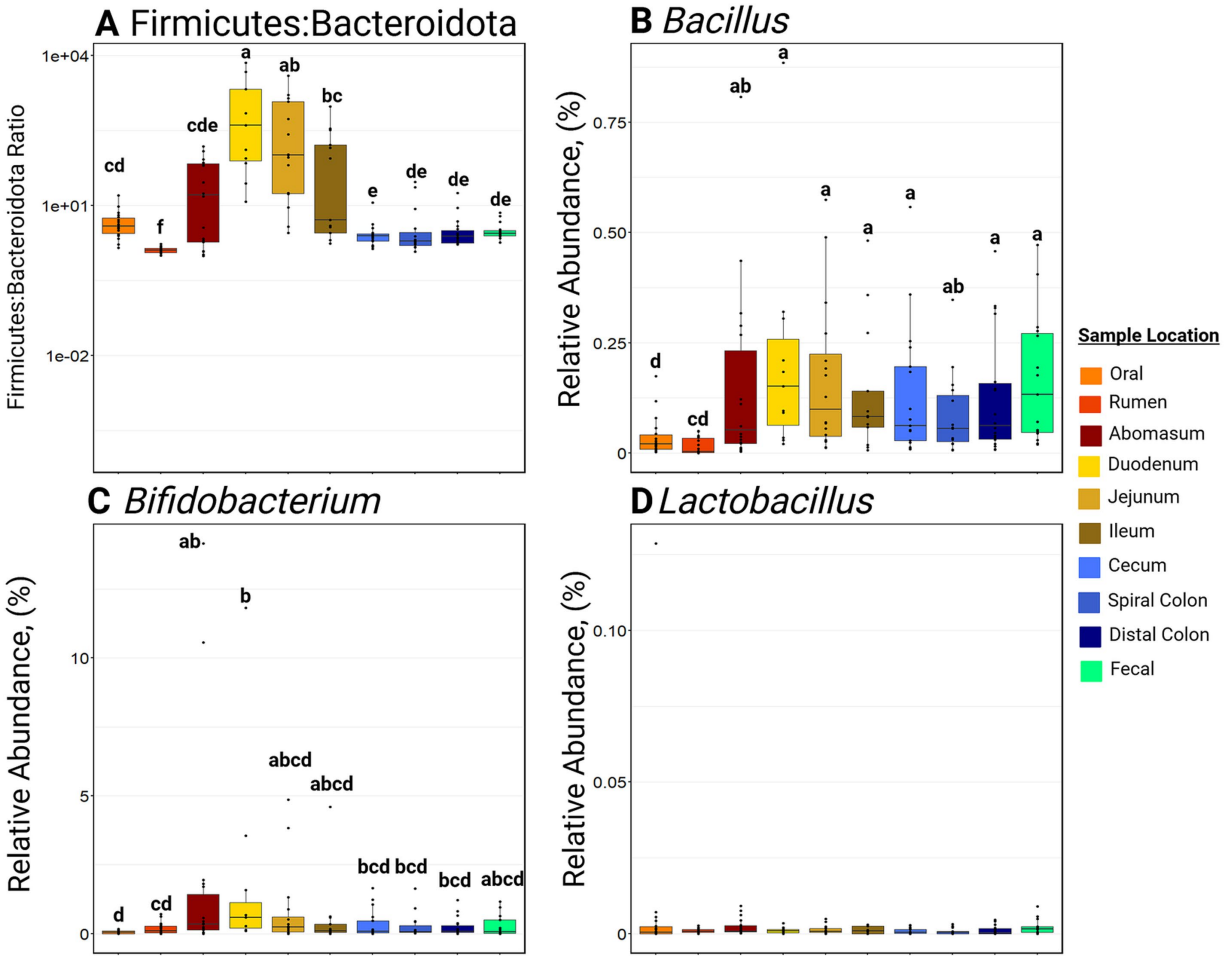
Boxplots of relative abundance of bacteria commonly associated with a healthy gastrointestinal tract. Panel **(A)** represents the Firmicutes to Bacteroidota ratio at various sites. Panel **(B)** represents the relative abundance of Bacillus at various sites. Panel **(C)** represents the relative abundance of Bifidobacterium at various sites. Panel **(D)** represents the relative abundance of Lactobacillus at various sites. Within a plot, boxes with different superscripts (abcdefg) differ by a pairwise Wilcoxon rank sum test (*p* ≤ 0.05). Plots were created in R, and legends were added using BioRender.com.

Samples collected from the small intestine had greater F:B ratios (*p* ≤ 0.05) compared to other locations in the GIT (Figure 5). Notably, the rumen had the lowest (*p* ≤ 0.001) F:B ratio of all the GIT locations. *Bifidobacterium* RA was greatest in the abomasum and duodenum compared to the oral and rumen samples (*p* ≤ 0.05; Figure 5). *Bifidobacterium* RA was also greater in the small intestine and hindgut than in the rumen (*p* ≤ 0.05). *Bacillus* RA was greater in the abomasum, duodenum, jejunum, ileum, cecum, spiral colon, distal colon, and feces than in the rumen (*p* ≤ 0.05). *Lactobacillus* RA was not significantly different among sample sites and was generally in low abundance along the entire GIT (RA ≤ 0.05%).

**Figure 6:**
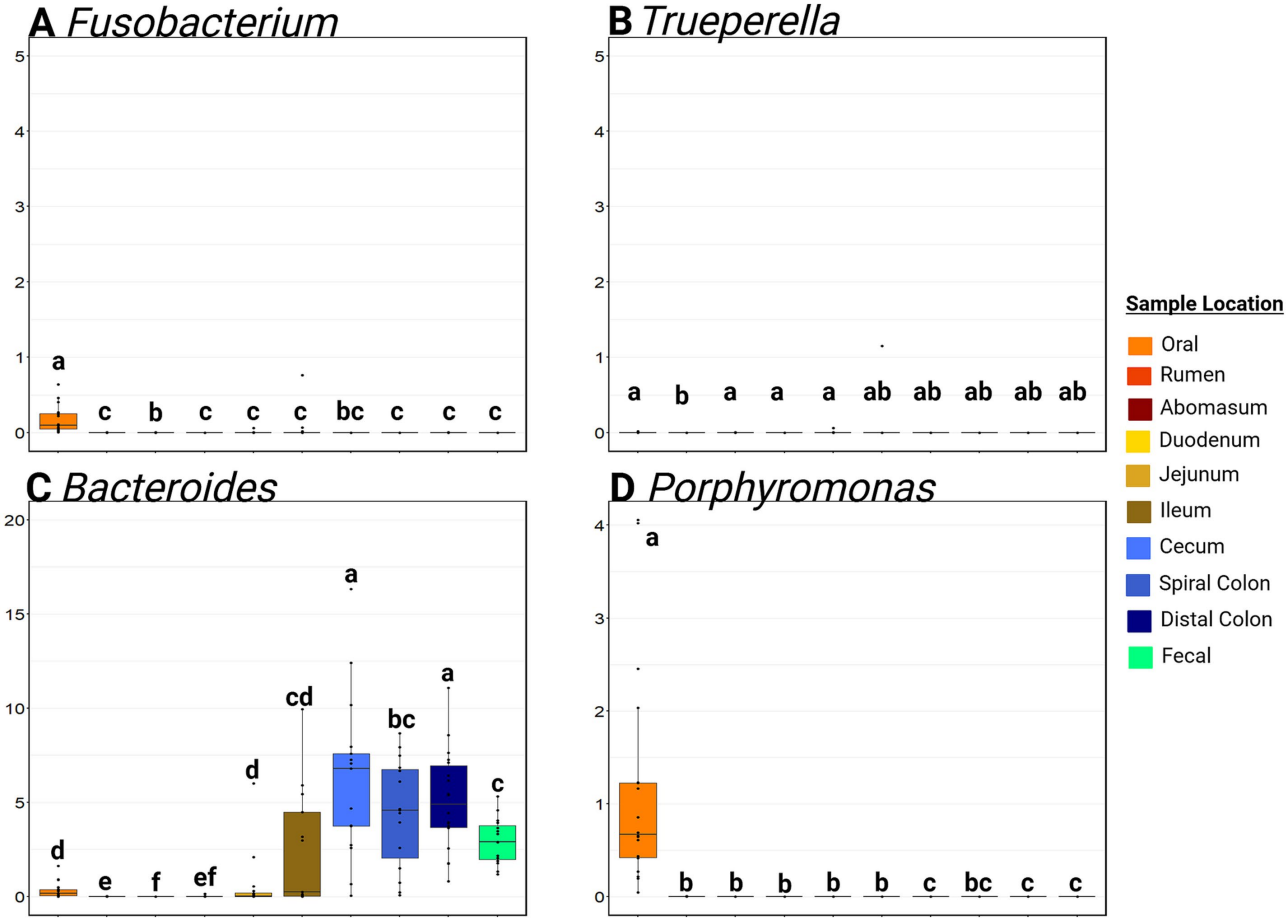
Boxplots of the relative abundance along the gastrointestinal tract of genera associated with liver abscesses. Panel **(A)** represents the relative abundance of Fusobacterium at various sites. Panel **(B)** represents the relative abundance of Truperella at various sites. Panel **(C)** represents the relative abundance of Bacteroides at various sites. Panel **(D)** represents the relative abundance of Porphyromonas at various sites. Within a plot, boxes with different superscripts (abcdef) differ by a pairwise Wilcoxon rank sum test (*p* ≤ 0.05). Plots were created in R, and legends were added using BioRender.com.

*Fusobacterium* RA was significantly greater in oral samples than anywhere else in the GIT (mean RA in oral samples = 0.17, 95% CI: 0.08, 0.26%; mean RA in other samples = 0.02, 95%CI: 0.00, 0.09%; *p* ≤ 0.01; Figure 6). The RA of *Trueperella* was extremely low in all GIT samples. *Bacteroides* was detected at a greater RA (*p* ≤ 0.05) in the hindgut compared to the small intestine and foregut (*p* > 0.05) from the hindgut (average hindgut RA = 5.24, 95%CI: 2.84, 8.33%; average small intestine RA = 1.0, 95%CI: 0.00, 4.41%; average foregut RA = 0.11, 95%CI: 0.00, 0.53%). *Porphyromonas* RA was greatest in the oral samples (*p* ≤ 0.05) compared to all other sample types. The rumen, abomasum, duodenum, jejunum, and ileum all had low RA of *Porphyromonas* (<0.01% RA), and this genus was not detected in the cecum, distal colon, feces, or liver abscess samples. *Moraxella* was commonly detected in the mouth, and *Escherichia-Shigella* was most often detected in the small intestine (Supplementary Figure S6). Of note, *Salmonella* was not detected in any sample. The oral samples contained a much higher RA of Gamma-Proteobacteria than any other sample site (*p* ≤ 0.05).

### 3.6 Core analysis

Core genera were evaluated throughout the GIT and by region within the GIT. For all sample types, six genera (*Olsenella, Methanobrevibacter, [Eubacterium] coprostanoligenes group, [Ruminococcus] gauvreauii group, unclassified Lachnospiraceae, Lachnospiraceae NK3A20 group, Family XII AD3011 group, Romboutsia, Ruminococcus,* and *Turicibacter*) were detected at 0.1% RA in 90% of the samples (Supplementary Figure S7). For the more anterior GIT locations (mouth, rumen, and abomasum), only *Olsenella* was identified as a core genus (0.1% RA in these samples). However, the separation of oral samples from the rumen and abomasum resulted in 15 and 29 genera, respectively, as core inhabitants. Similarly, small and large intestine samples had several core genera, with 22 genera in the small intestine and 39 genera in the large intestine identified in at least 0.1% RA in 90% or more of the samples, respectively.

## 4 Discussion

This study provides a more detailed investigation of the biogeography of bovine GIT than other studies conducted to date. Rarefaction of our sequencing data suggested that sequencing depths commonly used previously (e.g., 30,000–50,000 ASVs/sample) are likely insufficient to adequately characterize all taxa found in the mucosal microbiomes of the GIT. Clear differences in microbial abundance, diversity, and community composition are evident at different GIT locations. Unfortunately, while rumen and fecal samples are easily obtained, neither can be used as proxies for appropriately representing the microbiomes of other GIT locations, which has been described previously (de Oliveira et al., 2013; Mao et al., 2015; Lin et al., 2023). Therefore, researchers should be cautious to not overgeneralize when samples are only evaluated from a single GIT site.

A sharp drop in microbial abundance observed in the small intestine contrasted with the much higher microbial abundance of the cecum and colon. This finding, combined with the marked community structure differences observed in hindgut samples, substantiates the conclusion that the hindgut represents a distinctly different microbial community from the foregut. This distinction implies equally different population interactions and metabolic dynamics that may be important to further characterize. Our results also indicate that phylum-level data likely oversimplify the complex community structures of the GIT, and more specific (lower) taxonomic ranks should be utilized, potentially allowing greater elucidation of microbial community composition and function. Additionally, taxa previously identified as beneficial or harmful exist throughout the entire GIT of cattle but typically in very low abundance. Therefore, targeted approaches will likely be necessary to study these potentially important bacteria.

Analysis of microbial abundance quantification as estimated using qPCR of the 16S rRNA gene revealed a markedly lower microbial abundance in the small intestine, which has not been investigated extensively in cattle. However, other research has previously shown that small intestine microbial communities exhibit lower diversity and richness than other GIT regions (Malmuthuge et al., 2014; Durso et al., 2017; Plaizier et al., 2020). Specifically, several authors have documented lower richness in the small intestine relative to the large intestine. Plaizier et al. (2020) additionally reported greater richness and diversity in the cecum and rectum than the rumen, aligning with our findings.

Biologically, it is logical that the rumen and large intestine of cattle harbor more abundant and diverse microbial communities, given their capacity for fermentation and active utilization of fiber and other feed substrates. However, a significant environmental pH transition occurs along the GIT, from the abomasum’s acidic conditions to the jejunum’s neutral environment, which is quickly counteracted by the introduction of bile in the duodenum. This rapid influx of bile salts may perturb bacterial populations, while antimicrobial peptides and increased ingesta flow rates could impede bacterial proliferation (Church, 1988; McCallum and Tropini, 2023).

Apart from the oral cavity, similar microbial populations were noted in anatomically neighboring GIT sites. As reported previously, the oral cavity’s unique environmental exposures likely contribute to its different microbial community structure (Borsanelli et al., 2022). Studies investigating microbial community composition in different GIT regions have yielded varying results. Malmuthuge et al. (2014) reported that microbial communities of the rumen and large intestine of 3-week-old calves were more similar than those in the small intestine. Conversely, Plaizier et al. (2020) found distinct microbial community structures in the rumen, small intestine, and large intestine of yearling Holstein steers. Our findings suggest that the foregut and small intestine communities are more similar than the large intestine sites, differing from the conclusions of Malmuthuge et al. (2014). However, the age differences between animals in these studies complicate direct comparisons. That study focused on 3-week-old calves that lacked a fully developed rumen in terms of anatomy, physiology, and microbial community. Consequently, our results would be more comparable to studies investigating mature animals fed high-grain diets, which, unfortunately, are lacking in the current literature.

Firmicutes and Bacteroidota dominated in the rumen and the hindgut, consistent with a meta-analysis of 52 studies (Holman and Gzyl, 2019). However, evaluating microbial composition at the phylum level likely provides incomplete insights, as it overlooks nuances discernable only at more granular taxonomic levels (Walker and Hoyles, 2023). At the family level, distinctions were evident across the GIT. For example, *Lachnospiraceae* exhibited high abundance within the phylum Firmicutes across all sample types. In contrast, *Peptostreptococcaceae* was observed in low abundance in the mouth, rumen, abomasum, and duodenum but in high abundance in the jejunum, ileum, cecum, spiral colon, distal colon, and fecal samples. Similarly, within Bacteroidota, *Prevotellaceae* was highly abundant in the rumen but less so in the hindgut, where it appears to have been supplanted by *Bacteroidaceae*. The presence of these families aligns with previous observations (de Oliveira et al., 2013; Plaizier et al., 2020).

Actinobacteria have received less attention in microbiome studies compared to Firmicutes and Bacteroidota, with most investigations focusing solely on their high abundance in the small intestine (Plaizier et al., 2020) or exploring potential benefits of administering specific Actinobacteria strains as probiotics (Binda et al., 2018). However, there is a notable gap in data regarding the specific microbial community compositions at lower taxonomic levels in different GIT regions of feedlot cattle. With sequencing technologies’ growing affordability and advancements in data classification, future research should delve deeper into these nuances and explore the subtle differences in GIT microbial community structures. Moreover, investigations into the transcriptome and metabolome will likely offer substantial insights into functional characteristics of the microbiome that DNA characterizations cannot fully capture alone.

While understanding the dynamics of microbial community structures is crucial, certain bacteria are believed to offer benefits when administered as a probiotic. Typically, probiotics containing “good gut” bacteria are suggested to promote gastrointestinal health by directly modifying tight junction protein regulation or limiting the colonization of pathogens (Krehbiel et al., 2003; Welch et al., 2022). *Bifidobacterium, Bacillus,* and *Lactobacillus* are three common genera discussed as “good gut” bacteria (Binda et al., 2018; Smock et al., 2020; Cull et al., 2022). However, previous studies have primarily investigated the effects of supplementing these bacteria in the diet as probiotics. This study characterized naturally occurring bacterial communities, where these genera consistently represent less than 2.5% relative abundance, on average, throughout the GIT. The literature suggests significant performance benefits can be realized when they are supplemented in high abundance (Binda et al., 2018; Fuerniss et al., 2022b; Word et al., 2022). The ratio of abundances for Firmicutes to Bacteroidota (F:B ratio) is a standard metric used to assess the health of GIT microbial community composition in people (Stojanov et al., 2020; McCallum and Tropini, 2023). However, there is limited understanding of what constitutes a “healthy” F:B ratio in cattle. In human medicine, the F:B ratio is classically linked to obesity and tends to increase as people age (Magne et al., 2020; Vaiserman et al., 2020). Typically a ratio of 1.5:1 is reported in humans (Magne et al., 2020; Vaiserman et al., 2020) but a ratio of 2.33:1 has been reported in the feces of cattle (Zhang et al., 2021). However, Zhang et al. (2021) looked at calves in a feedlot and on pasture. Since the F:B ratio has been shown to increase from high energy diet consumption (Magne et al., 2020), feedlot cattle consuming high concentrate rations might show a greater F:B ratio. Furthermore, the F:B ratio has typically only been explored in feces, but the increase in the ratio in other compartments is logical. Specifically, an increase in the F:B ratio in the small intestine likely stems from an increased need for Firmicutes to digest a high energy ration.

Liver abscesses in cattle occur most commonly due to bacterial translocation from the gut into the hepatic portal circulation (Nagaraja and Chengappa, 1998; Broadway et al., 2024). Recent microbiome studies of liver abscesses from feedlot cattle suggest these lesions have highly polymicrobial communities (Fuerniss et al., 2022a; Pinnell et al., 2022). Historical dogma suggested that the ruminal lesions were the dominant source for bacteria seeding these abscesses, but several authors have recently identified a significant prevalence of abscesses with a high abundance of *Bacteroidaceae*, suggesting that a source linked to more distal portions of the GIT. This is supported by a recent report by Pinnell et al. (2023), who found a higher abundance of *Bacteroidaceae* in colon microbial communities compared to the rumen and ileum. This finding aligns with the results of the current study. Pinnell et al. (2023) also reported a subset of highly poly microbial abscess samples containing the family *Porphyromonadaceae*. In this study, *Porphyromonas* was highly abundant in the oral samples but nowhere else along the GIT.

## Data Availability

The original contributions presented in the study are publicly available. This data can be found at: DOI: 10.5281/zenodo.14231575. The code and instructions for the bioinformatic and statistical analyses can be found at this GitHub repository: https://github.com/Microbial-Ecology-Group/Full_GI_manuscript_code. The names of the repository/repositories and accession number(s) can be found at: https://www.ncbi.nlm.nih.gov/, PRJNA1108714.

## Supplementary material

SUPPLEMENTARY FIGURE S1 Boxplot of total microbial abundance between cohorts. Plots were created in R, and legends were added using BioRender.com.

SUPPLEMENTARY FIGURE S2 Rarefaction of each sample from a depth of 0 to 1,000,000 reads per sample. Plots were created in R, and legends were added using BioRender.com.

SUPPLEMENTARY FIGURE S3 Boxplot of observed richness and shannons diversity index between harvest cohorts. Plots were created in R, and legends were added using BioRender.com.

SUPPLEMENTARY FIGURE S4 Ordination of community structure by harvest cohort. Non-metric multidirectional scaling (NMDS) of generalized Unifrac distances illustrate differences in microbial community structure between sample sites. Ellipses represent the 95% confidence intervals for the group mean values. Plots were created in R, and legends were added using BioRender.com.

SUPPLEMENTARY FIGURE S5 Relevant phyla by family within cohort. **(A)** represents families belonging to the Firmicutes phyla split by cohort. **(B)** represents families belonging to the Bacteroidota phyla split by cohort. **(C)** represents families belonging to the Actinobacteriota phyla split by cohort. Plots were created in R, and legends were added using BioRender.com.

SUPPLEMENTARY FIGURE S6 The class Gamma-Proteobacteria, separated by genus and sample location. *Moraxella* was highly abundant in the oral samples, while *Escherichia-shigella* was the most abundant pathogen in the GIT with the largest RA detected in the small intestine. Plots were created in R, and legends were added using BioRender.com.

SUPPLEMENTARY FIGURE S7 Dots represent sample means and the lines indicate a 95% confidence interval. Plots were created in R, and legends were added using BioRender.com.
