# Supplementary figures and images for: The biogeography of gastrointestinal mucosal microbiota of beef cattle at harvest
Source: Front Microbiol. 2024 Dec 9;15:1490882. doi: 10.3389/fmicb.2024.1490882 (PMC11663860; doi:10.3389/fmicb.2024.1490882)

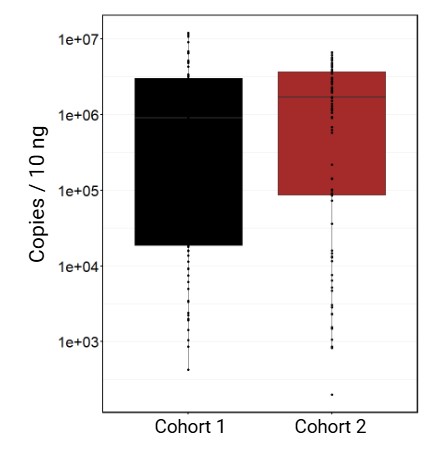

Supplement: SUPPLEMENTARY FIGURE S1 — Boxplot of total microbial abundance between cohorts. Plots were created in R, and legends were added using BioRender.com. [file Image_1.JPEG]

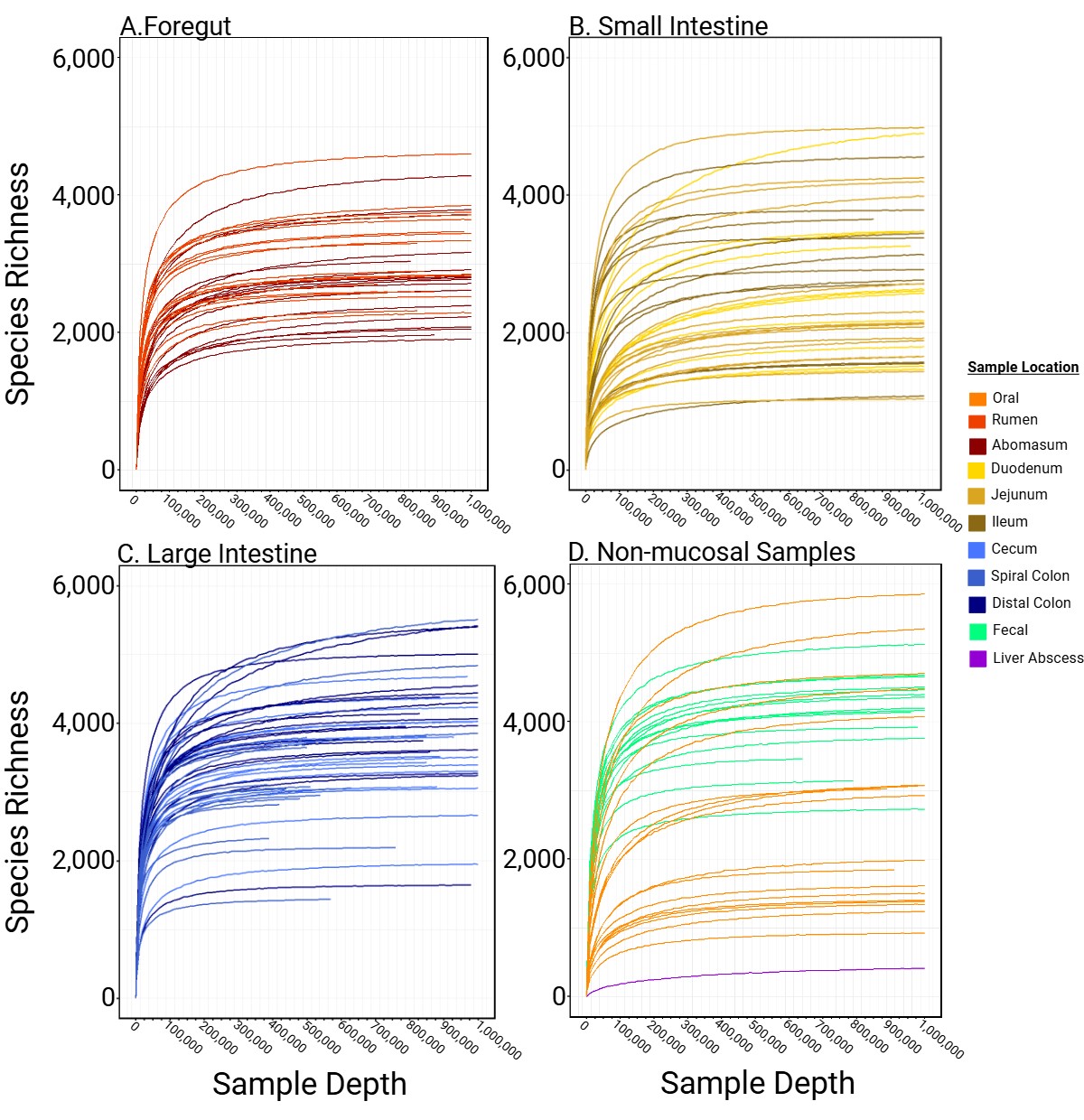

Supplement: SUPPLEMENTARY FIGURE S2 — Rarefaction of each sample from a depth of 0 to 1,000,000 reads per sample. Plots were created in R, and legends were added using BioRender.com. [file Image_2.JPEG]

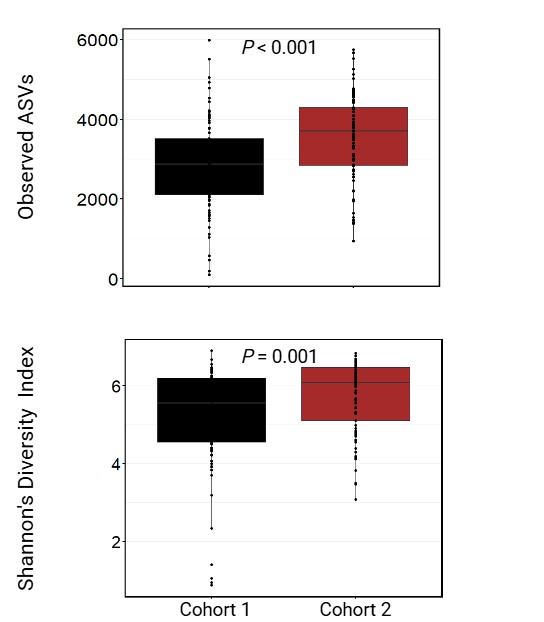

Supplement: SUPPLEMENTARY FIGURE S3 — Boxplot of observed richness and shannons diversity index between harvest cohorts. Plots were created in R, and legends were added using BioRender.com. [file Image_3.JPEG]

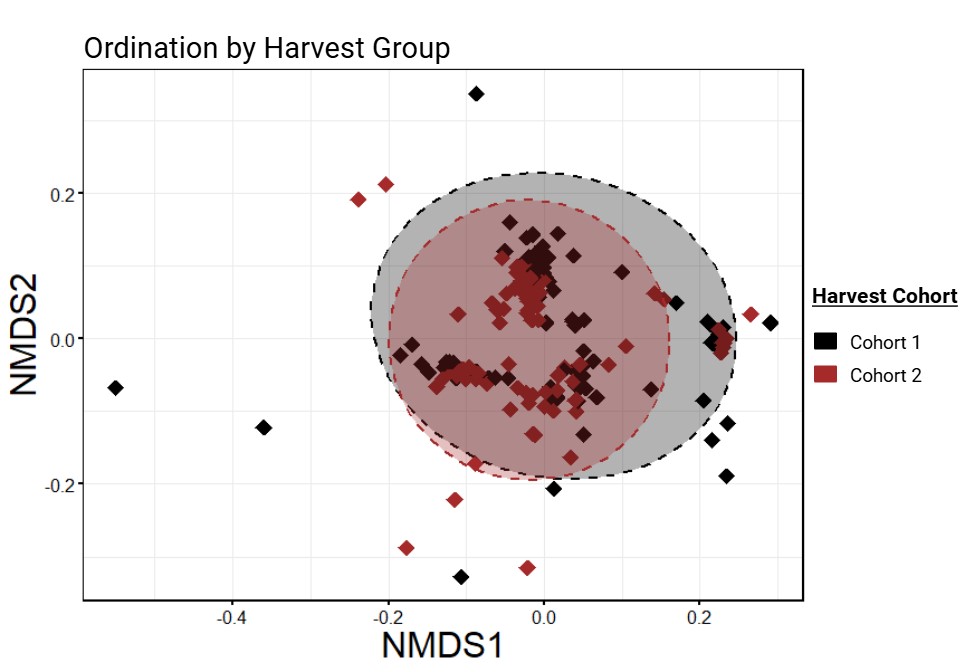

Supplement: SUPPLEMENTARY FIGURE S4 — Ordination of community structure by harvest cohort. Non-metric multidirectional scaling (NMDS) of generalized Unifrac distances illustrate differences in microbial community structure between sample sites. Ellipses represent the 95% confidence intervals for the group mean values. Plots were created in R, and legends were added using BioRender.com. [file Image_4.JPEG]

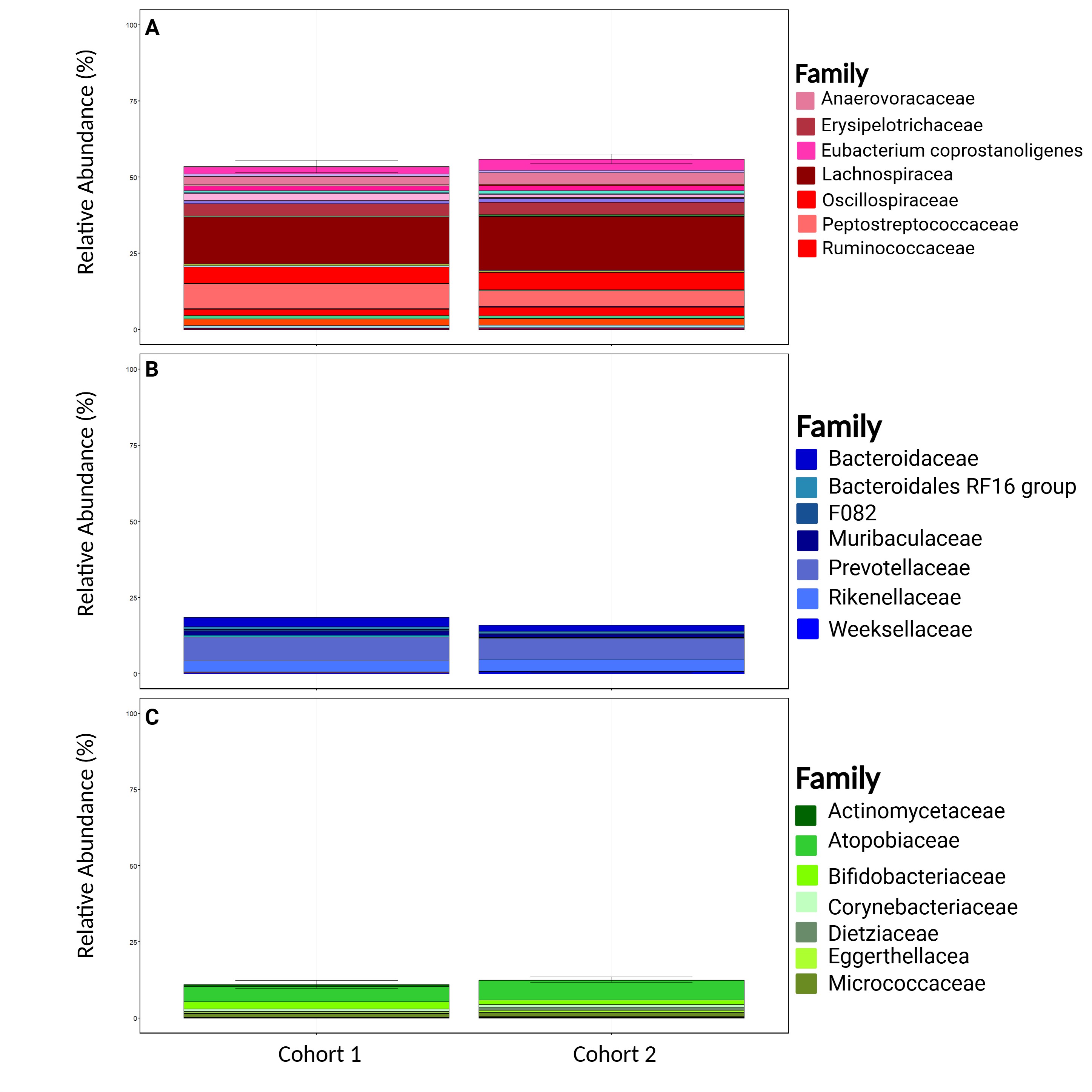

Supplement: SUPPLEMENTARY FIGURE S5 — Relevant phyla by family within cohort. (A) represents families belonging to the Firmicutes phyla split by cohort. (B) represents families belonging to the Bacteroidota phyla split by cohort. (C) represents families belonging to the Actinobacteriota phyla split by cohort. Plots were created in R, and legends were added using BioRender.com. [file Image_5.JPEG]

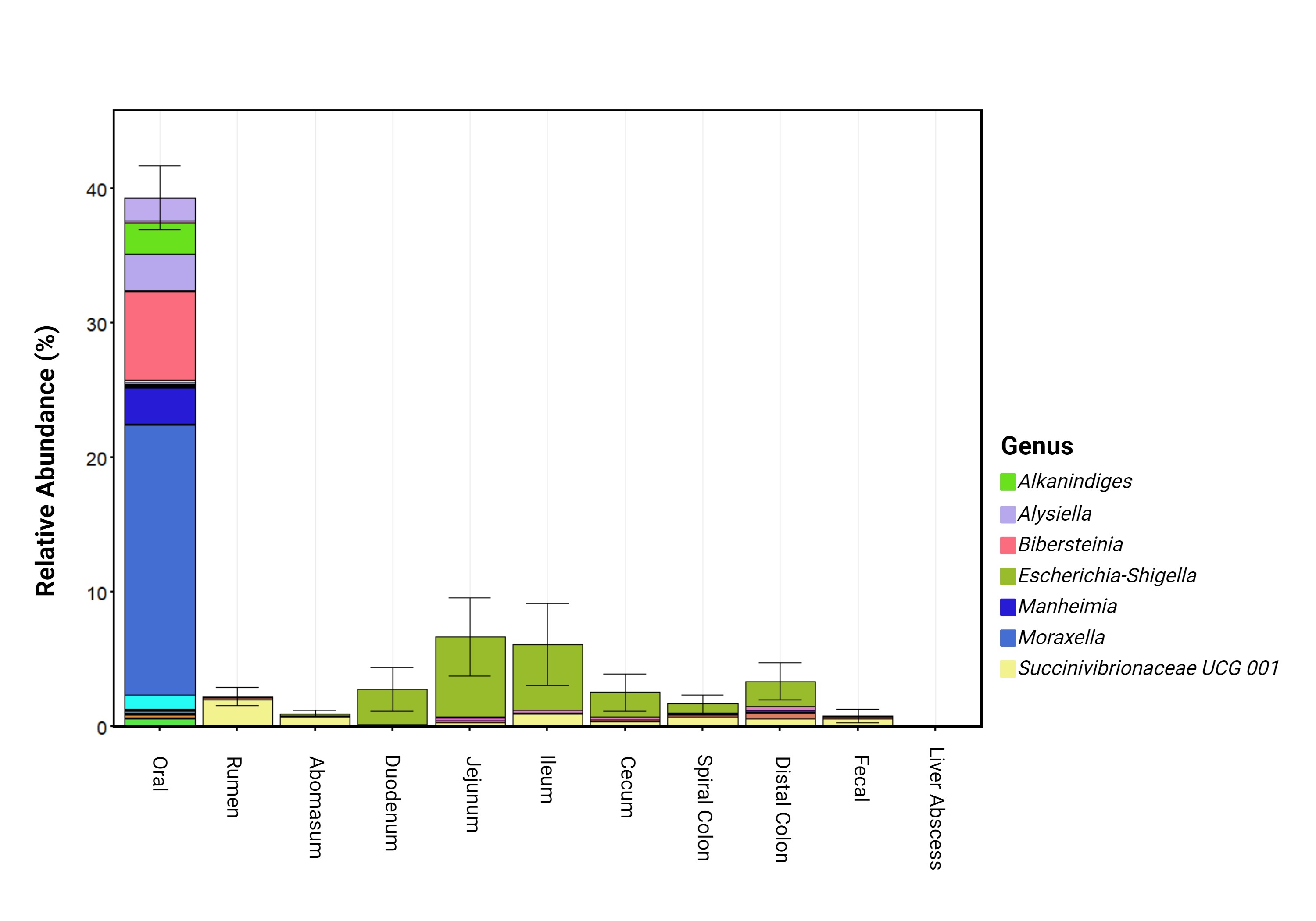

Supplement: SUPPLEMENTARY FIGURE S6 — The class Gamma-Proteobacteria, separated by genus and sample location. Moraxella was highly abundant in the oral samples, while Escherichia-shigella was the most abundant pathogen in the GIT with the largest RA detected in the small intestine. Plots were created in R, and legends were added using BioRender.com. [file Image_6.JPEG]

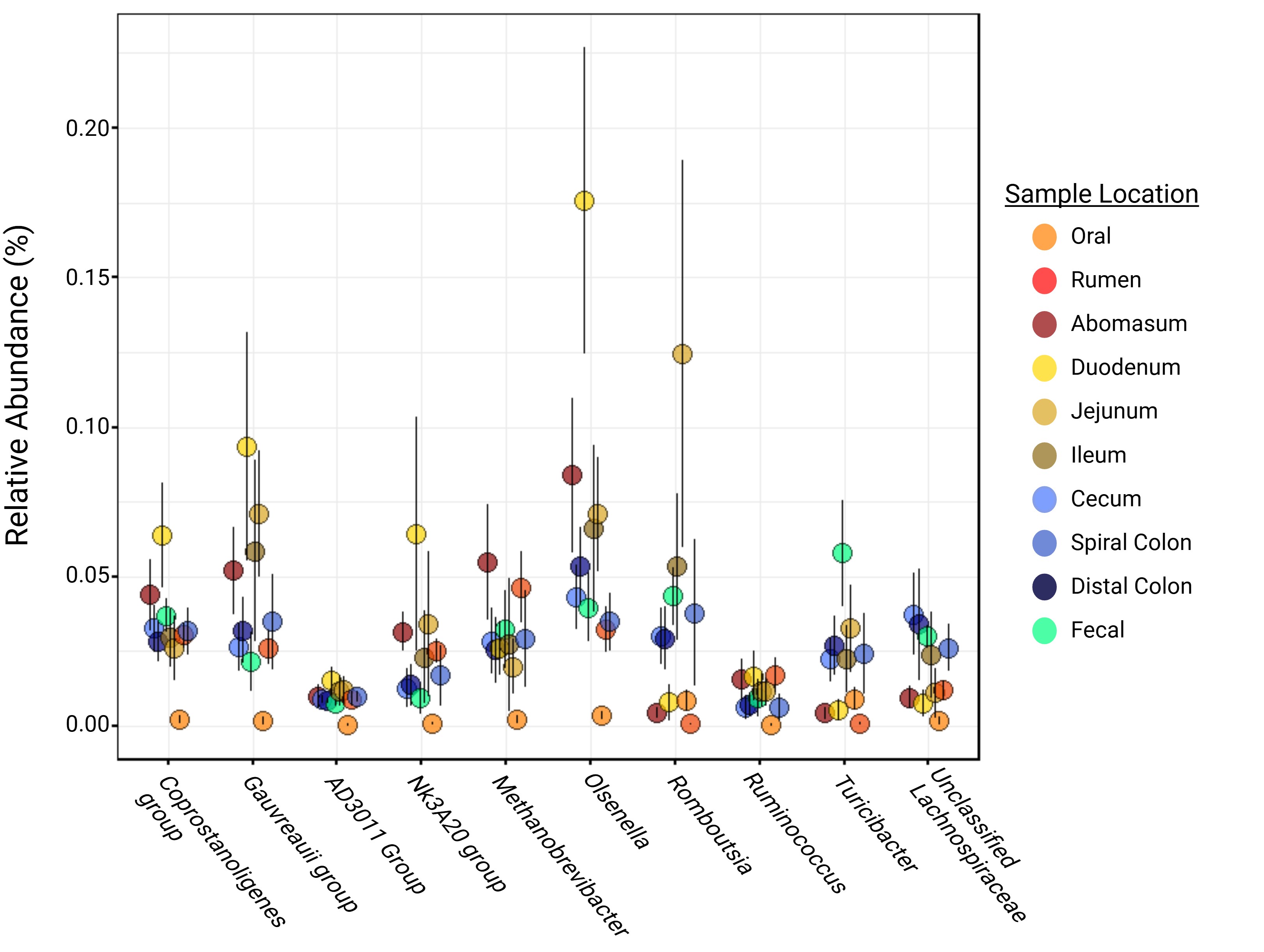

Supplement: SUPPLEMENTARY FIGURE S7 — Dots represent sample means and the lines indicate a 95% confidence interval. Plots were created in R, and legends were added using BioRender.com. [file Image_7.JPEG]
