# Supplementary material for: The biogeography of gastrointestinal mucosal microbiota of beef cattle at harvest
Source: Front Microbiol. 2024 Dec 9;15:1490882. doi: 10.3389/fmicb.2024.1490882 (PMC11663860; doi:10.3389/fmicb.2024.1490882)
Supplement: Supplementary file 8 [file Data_Sheet_1.pdf]

**Table S1.** Diet composition of all rations fed to cattle on a dry matter basis.

| Ingredient | Starting Ration, % | Finishing Ration, % |
|---|---|---|
| Corn grain, Flaked | 28.54 | 54.10 |
| Corn Stalks | 19.00 | 7.76 |
| Sweetbran[1] | 42.00 | 29.43 |
| Molasses | 7.00 | 3.49 |
| Corn Oil | 0.00 | 1.92 |
| Mineral Supplement | 3.46 | 3.30 |

[1]Cargill Corn Milling

**Table S2.** Classification efficiency by taxonomic rank.

| Rank | Percentage[1] |
|---|---|
| Phylum | 99.35 |
| Class | 99.34 |
| Order | 99.31 |
| Family | 99.07 |
| Genus | 93.18 |

[1]Percent of samples after the initial trim which were classified at each taxonomic rank.
